# Western Medical Appointments

**Published:** 1838

**Authors:** 


### Art. IV.—Western Medical Appointments.

Since our last number, the following appointments, or acceptances, have taken place in the West.

*Medical College of Ohio*—Dr. R. D. Mussey of Dartmouth College, to the chair of Surgery.

*Transylvania University*—Dr. T. D. Mitchell transferred from the chair of Chemistry to that of Materia Medica and Therapeutics, *vice* Dr. Short, resigned. Dr. Robert Peter appointed professor of Chemistry. Dr. Nathan R. Smith, late professor of Surgery in the University of Maryland appointed to the chair of Theory and Practice, rendered vacant by the death of Dr. Eberle. Dr. S. will continue to reside in Baltimore.

*Louisville Medical Institute*—Dr. Charles W. Short of Transylvania University, appointed to the chair of Materia Medica and Botany.

*Willoughby University of Lake Erie*—Dr. J. De La Mater, of the Fairfield school New York to the chair of Theory and Practice, *vice* Dr. Piexotto, resigned.

*Medical College of Louisiana*—We believe there were lately some vacancies in this institution. Whether they have been filled we are unable to state, as our friends in this institution do not keep us advised of its actual condition.

*Medical Department of the Cincinnati College*—It is worthy of remark that but one resignation and one new appointment have taken place in this institution since its commencement. Perhaps no school in the Union has undergone, in this respect, so few changes, while in its forming state. At present every thing indicates that it has a consolidated and permanent Faculty. D.

